# Changes in Salivary Interleukin-1 Beta Levels Following a Care Worker-Centered Oral Health Care Program Among Residents of Long-Term Care Facilities

**DOI:** 10.3390/healthcare14162667

**Published:** 2026-08-21

**Authors:** Hee-Jung Lim, Da-Eun Kim, Se-Eun Jang, Ki-Hyun Yoon, Jun-Yeong Kwon

**Affiliations:** 1 Department of Dental Hygiene, College of Health Science, Eulji University, Seongnam 13135, Republic of Korea; cindy-1109@eulji.ac.kr (H.-J.L.); prde0110@naver.com (D.-E.K.); 2 Department of Food and Nutrition, Bio Convergence College, Eulji University, Seongnam 13135, Republic of Korea; sejang@eulji.ac.kr (S.-E.J.); josh0520@naver.com (K.-H.Y.)

**Keywords:** long-term care facilities, care workers, oral healthcare, older adults, salivary biomarkers, interleukin-1 beta

## Abstract

**Highlights:**

**What are the main findings?**

- A care worker-centered oral healthcare program significantly reduced salivary interleukin-1 beta (IL-1β) concentrations among older adults residing in long-term care facilities (LTCFs).
- The effectiveness of the intervention was objectively confirmed using salivary IL-1β rather than relying solely on clinical oral health indices.

**What are the implications of the main findings?**

- Care worker-delivered oral healthcare represents a practical and sustainable strategy for improving oral health among residents of LTCFs.
- Salivary IL-1β may serve as a useful biomarker for evaluating the effectiveness of oral healthcare interventions in institutionalized older adults.

**Abstract:**

**Background/Objectives:** Older adults residing in long-term care facilities (LTCFs) are at increased risk of poor oral health because of functional dependence and limited access to professional oral healthcare. Although oral healthcare education programs for care workers improve knowledge and performance, evidence of their effects on residents’ biological oral health outcomes remains limited. This study evaluated the effects of a care worker-centered oral healthcare program on oral inflammatory status in LTCF residents using salivary interleukin-1 beta (IL-1β) as an objective biomarker. **Methods:** A single-group pretest–posttest quasi-experimental study was conducted in two LTCFs in the Republic of Korea. Forty care workers completed a structured oral healthcare education program and provided daily oral healthcare to residents for 4 weeks. Saliva samples were collected before and after the intervention, and salivary IL-1β concentrations were measured using enzyme-linked immunosorbent assay. Changes in IL-1β concentrations were analyzed using the Wilcoxon signed-rank, Mann–Whitney U, and Kruskal–Wallis tests. **Results:** Thirty-seven residents completed the study. Median salivary IL-1β concentrations significantly decreased from 19.33 pg/mL (interquartile range [IQR], 11.49–34.66) at baseline to 10.78 pg/mL (IQR, 0.65–33.64) after the intervention (Z = −3.764, *p* = 0.001). Greater reductions were observed among residents with greater functional dependence, whereas limited communication ability or oral care refusal was associated with minimal improvement. **Conclusions:** A care worker-centered oral healthcare program significantly reduced salivary IL-1β concentrations among older adults residing in LTCFs. Findings support care worker training and highlight the use of salivary IL-1β as an objective biomarker for evaluating oral healthcare interventions.

## 1. Introduction

The global population is aging rapidly, leading to a continuous increase in the demand for long-term care (LTC) services [1,2]. In particular, South Korea officially became a super-aged society in 2025 and is among the countries experiencing the fastest growth in the proportion of older adults worldwide [2,3]. Consequently, the utilization of long-term care facilities (LTCFs) and long-term care hospitals has steadily increased [4,5], accompanied by growing social attention to the health management and care of older adults. Beyond extending life expectancy, integrated healthcare aimed at promoting healthy aging and maintaining quality of life has become increasingly important [1].

Older adults residing in LTCFs often experience physical and cognitive impairments accompanied by multiple chronic conditions [6]. Because of limitations in activities of daily living (ADLs), they rely heavily on care workers for essential daily activities, including eating and personal hygiene [7]. This functional dependency directly affects their ability to maintain oral hygiene independently [8], placing them at high risk of progressive deterioration in oral health because of inadequate self-care [9].

Residents of LTCFs commonly present with poor oral health, including dental plaque accumulation, periodontal disease, dental caries, tooth loss, xerostomia, and inappropriate denture use [6,10]. Additionally, age-related deterioration of the oral and perioral muscles impairs mastication and swallowing function [11], whereas increased oral microbial colonization may contribute to aspiration pneumonia, malnutrition, and systemic inflammatory responses [12]. Therefore, maintaining oral health in LTCF residents is essential for preventing oral diseases, preserving systemic health, and improving quality of life [9,13].

However, most LTCFs do not employ oral healthcare professionals, such as dentists or dental hygienists, on a full-time basis. Consequently, routine oral care for residents is primarily provided by care workers, including certified care assistants [14]. In South Korea, the implementation of the Act on Integrated Support for Community Care Including Medical and Long-Term Care Services has further emphasized the importance of oral healthcare for older adults by incorporating home-based oral healthcare into integrated community care services [15,16]. Nevertheless, institutional support for oral healthcare delivery in LTCFs remains insufficient, particularly regarding service delivery systems, professional roles, scope of practice, and standardized protocols [17]. Under these circumstances, care workers play a pivotal role in providing routine oral care and are the healthcare personnel who interact most frequently with residents, making them central to maintaining the quality of daily oral hygiene.

Previous studies have shown that although care workers recognize the importance of oral health, they often receive insufficient education on oral disease prevention, denture care, oral care for residents with dysphagia, and routine oral hygiene management [18,19]. Accordingly, various oral health education programs have been developed for care workers, and these programs improve their oral health-related knowledge, attitudes, and caregiving practices [20,21]. However, most previous studies have focused primarily on evaluating educational outcomes among care workers, such as improvements in knowledge, attitudes, and performance [20,21]. Evidence demonstrating whether these educational interventions lead to measurable improvements in the oral health of LTCF residents remains limited. Therefore, objective evaluation of the effectiveness of care worker-centered oral healthcare programs using biological indicators that reflect actual oral health outcomes in residents is needed.

Saliva has recently gained attention as a useful diagnostic medium for assessing oral health because samples can be collected non-invasively and repeatedly [22]. Among salivary biomarkers, interleukin-1β (IL-1β) is a representative pro-inflammatory cytokine involved in periodontal inflammation and tissue destruction [22,23]. Salivary IL-1β concentrations are closely associated with periodontitis severity [24] and are sensitive biomarkers for monitoring changes following periodontal treatment and oral health interventions [22,23]. Therefore, salivary IL-1β may serve as a practical biological indicator for assessing oral inflammatory status in older adults residing in LTCFs, for whom comprehensive periodontal examinations are often difficult to perform.

Therefore, the present study compared salivary IL-1β concentrations before and after an oral healthcare intervention among older adults residing in LTCFs to objectively evaluate the effectiveness of a care worker-centered oral healthcare program in improving oral inflammatory status. By providing biological evidence of the program’s effectiveness, this study aims to support the implementation of evidence-based oral healthcare programs in LTCFs and contribute to the development of systematic oral healthcare services and educational programs for care workers.

## 2. Materials and Methods

### 2.1. Study Design

**Figure 1:**
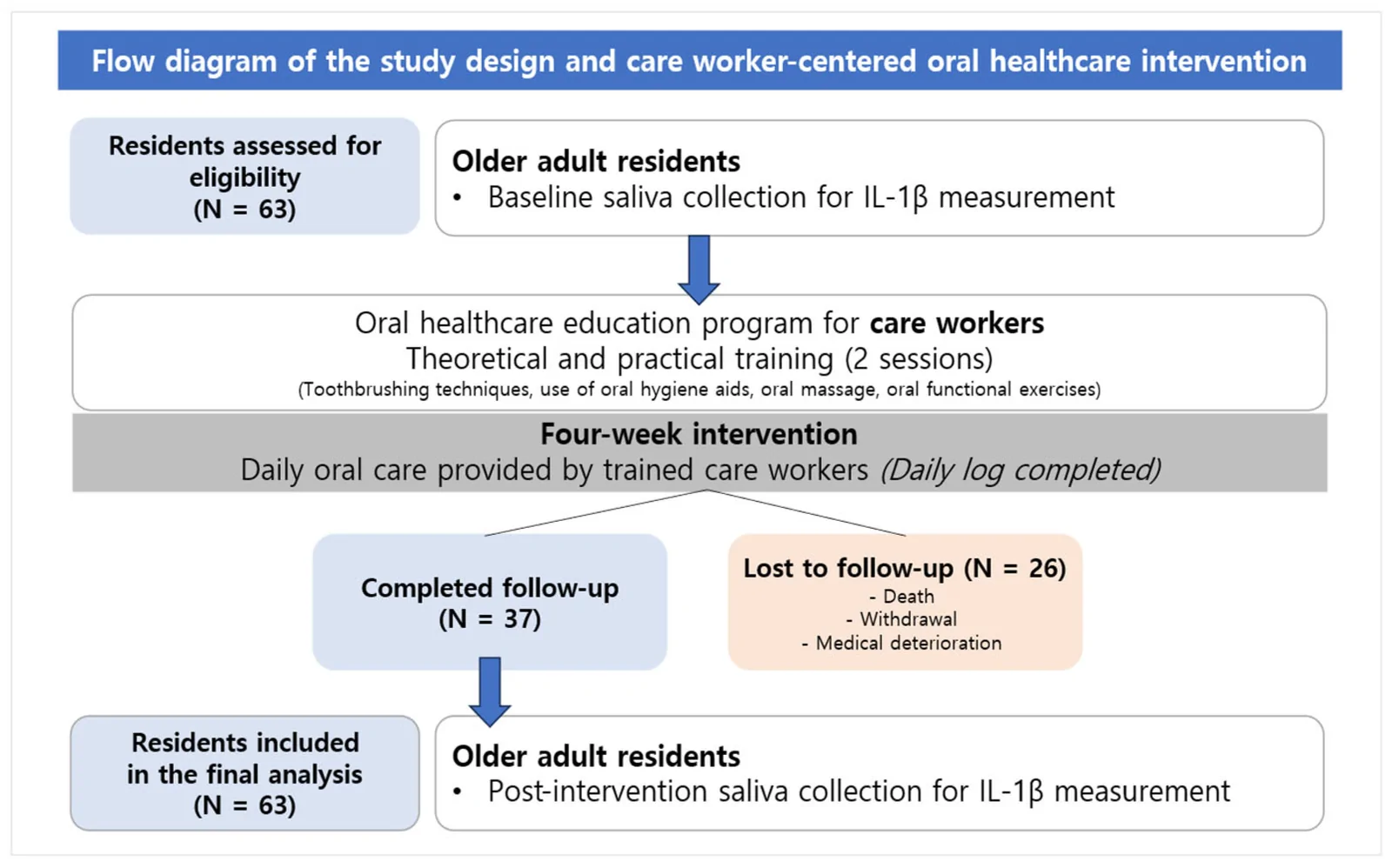
Flow diagram of the study design and intervention procedures.

This study employed a single-group pretest–posttest quasi-experimental design to evaluate the effects of a care worker-centered oral healthcare program on oral health-related biological indicators among older adults residing in LTCFs. The study was conducted over approximately 3 months, from 11 February to 6 May 2026, at two LTCFs located in K Province, Republic of Korea. The intervention was delivered to care workers employed at the participating LTCFs, whereas its effectiveness was evaluated among older adult residents who received daily oral care from the trained care workers. Following completion of the oral healthcare program, the care workers provided routine oral care to the residents for 4 consecutive weeks. To evaluate the effectiveness of the intervention, saliva samples were collected from the residents at baseline (before the intervention) and immediately after completion of the 4-week intervention. Salivary IL-1β concentrations were subsequently analyzed to objectively assess changes in oral inflammatory status. The overall study design and participant flow are illustrated in Figure 1. This study was approved by the Institutional Review Board (IRB) (IRB No. EUIRB2025-073). Written informed consent was obtained from participants or, when applicable, from their legally authorized representatives.

### 2.2. Participants

#### 2.2.1. Care Workers

The inclusion criteria for care workers were as follows: (1) certified care workers, nurse aides, or registered nurses employed at the participating LTCFs; (2) individuals responsible for providing routine oral care to residents; and (3) those who voluntarily agreed to participate in the study. A total of 40 care workers were enrolled. After completing the oral healthcare education program, the care workers provided routine oral healthcare to the residents for 4 consecutive weeks. To monitor intervention fidelity, the care workers completed a daily log documenting the oral healthcare provided throughout the intervention period.

#### 2.2.2. Residents

The inclusion criteria for residents were as follows: (1) aged 65 years or older; (2) residing in one of the participating LTCFs for at least 1 month; (3) able to provide saliva samples; and (4) providing written informed consent, either personally or through a legally authorized representative. Residents who declined to participate or were unable to complete the follow-up assessments during the intervention period were excluded. A total of 63 residents were initially enrolled. During the study period, 26 residents were lost to follow-up because of death, withdrawal of consent, or deterioration in their medical condition. Consequently, data from 37 residents were included in the final analysis. Baseline demographic, health-related, functional, and oral health-related characteristics were collected before the intervention. These included sex, age, long-term care grade, communication ability, mobility, ability to perform daily oral care, refusal of oral care, ability to keep the mouth open during oral care, ability to rinse, comorbidities (hypertension, diabetes mellitus, dementia, and osteoporosis), oral hygiene frequency, use of oral hygiene aids, diet consistency, and denture status.

#### 2.2.3. Sample Size

An a priori power analysis was performed using G*Power software (Version 3.1.9.7; Heinrich-Heine University Düsseldorf, Düsseldorf, Germany). Based on the expected within-subject changes following the intervention, a paired comparison design was assumed. A medium effect size (Cohen’s dz = 0.5), a two-tailed significance level (α = 0.05), and statistical power (1 − β = 0.95) were applied, resulting in a minimum required sample size of 45 participants. Although the sample size calculation was based on a paired comparison approach, the Wilcoxon signed-rank test was applied because the distribution of IL-1β concentrations did not satisfy the assumption of normality.

### 2.3. Oral Healthcare Intervention Program

The oral healthcare intervention program was jointly developed and delivered by an experienced dental hygienist and a professor in the Department of Dental Hygiene. The program consisted of standardized theoretical and practical training covering the relationship between common oral diseases and systemic diseases in older adults, toothbrushing techniques, oral moisturizing care, denture care, use of oral sponges, use of oral care moisturizing wipes, oral massage, and oral functional exercises. Practical training enabled care workers to directly practice each oral healthcare procedure under the guidance of the instructors. The education program was delivered in two sessions over a 2-week period, with each session lasting 2 h. To ensure standardized delivery of the intervention, all care workers received the same educational materials and practical training based on a standardized protocol developed by the research team.

Following completion of the education program, the trained care workers incorporated the standardized oral healthcare procedures into their routine daily oral care provided to residents according to each resident’s usual oral care schedule within the LTCFs. Care workers were instructed to apply the techniques practiced during the education sessions and to document the completion of each oral healthcare activity in a daily intervention log to monitor adherence.

Oral functional exercises, including breathing exercises using an elephant trumpet device and other oral motor exercises, were incorporated into the routine group activity programs of each LTCF. Standardized instructional videos developed by the research team were provided to facilitate consistent implementation, and residents were encouraged to perform the exercises once daily under the supervision of the LTCF staff.

Throughout the 4-week intervention period, members of the research team conducted regular on-site visits to monitor intervention fidelity. During these visits, the researchers reviewed the care workers’ intervention records, confirmed the implementation of the oral healthcare procedures, and provided individualized feedback regarding practical issues encountered during oral healthcare delivery, including bite-related incidents and difficulties in providing oral care for functionally dependent residents. All oral healthcare supplies required for the intervention were provided by the research team. The intervention protocol was designed to be incorporated into routine oral care practices without substantially increasing the daily workload of care workers.

### 2.4. Saliva Collection and Analysis

#### 2.4.1. Saliva Collection and Processing

Saliva samples were collected according to each resident’s ability to provide unstimulated saliva. For residents who provided unstimulated saliva voluntarily, trained dental hygienists collected saliva samples directly into sterile 50 mL centrifuge tubes. The collected saliva samples were centrifuged at 13,000 rpm for 10 min, after which the supernatant was carefully collected. Subsequently, 100 μL of the supernatant was diluted with 500 μL of phosphate-buffered saline (PBS) and stored at −70 °C until analysis. For residents who were unable to provide saliva voluntarily, a sterile cotton swab was placed in the oral cavity for approximately 1 min to absorb saliva. The swab was then transferred into a microcentrifuge tube containing 500 μL PBS and vortexed for 1 min to elute the saliva. The eluate was centrifuged at 13,000 rpm for 10 min, and the resulting supernatant was collected and stored at −70 °C until analysis. Overall, 11 residents (29.7%) provided unstimulated whole saliva, whereas 26 participants (70.3%) underwent saliva collection using oral swabs according to their ability to provide saliva.

#### 2.4.2. Measurement of Salivary IL-1β

Total protein concentrations of all saliva samples were determined using the Bradford assay. Before ELISA analysis, all saliva samples were normalized to the same total protein concentration (10 μg/mL) to minimize variability resulting from differences in saliva collection volume and sample dilution. Salivary concentrations of IL-1β were measured using the DuoSet™ ELISA Development System (R&D Systems, Minneapolis, MN, USA) according to the manufacturer’s instructions.

After completion of the reaction, absorbance was measured at 450 nm using a microplate reader. Absorbance values were corrected by subtracting the blank value, and a standard curve was generated using recombinant IL-1β standards according to the manufacturer’s instructions. IL-1β concentrations in each saliva sample were subsequently calculated from the corresponding standard curve. Blank wells and recombinant IL-1β standards were included in each assay according to the manufacturer’s instructions.

### 2.5. Statistical Analysis

The collected data were analyzed using SPSS version 25.0 (IBM Corp., Armonk, NY, USA). General characteristics of the participants were summarized using frequencies and percentages or means and standard deviations, as appropriate. Before statistical analysis, the data were screened for potential outliers and assessed for distributional characteristics. Normality of salivary IL-1β concentrations was evaluated using the Shapiro–Wilk test. Because the IL-1β concentrations were not normally distributed, non-parametric statistical tests were used. Changes in salivary IL-1β concentrations before and after the intervention were analyzed using the Wilcoxon signed-rank test. Differences in changes in IL-1β concentrations according to participants’ general characteristics were analyzed using the Mann–Whitney U test for comparisons between two groups and the Kruskal–Wallis test for comparisons among three or more groups. Pre- and post-intervention IL-1β concentrations in subgroup analyses are presented descriptively as mean ± standard deviation, whereas changes in IL-1β (ΔIL-1β), which were used for between-group comparisons, are presented as median (interquartile range). This approach was adopted because several subgroup categories contained very small sample sizes, limiting reliable estimation of quartiles. The primary outcome was the within-participant change in salivary IL-1β concentration before and after the intervention. Because of the relatively small final sample size and sparse subgroup categories, multivariable regression analyses were not performed, as such analyses were unlikely to produce stable estimates. No adjustment for multiple comparisons was applied because the subgroup analyses were conducted to provide descriptive comparisons across participant characteristics. A two-sided *p*-value < 0.05 was considered statistically significant.

## 3. Results

### 3.1. Characteristics of Study Participants

Participants’ general characteristics are presented in Table 1. Among the residents, females accounted for the majority (62.2%). Regarding long-term care insurance grades, grade 4 was the most prevalent category (40.5%). Most residents received oral care twice a day or less (91.9%). The oral care products used by residents included toothbrushes (86.5%) and denture cleansers (32.4%). Regarding oral function, 70.3% of residents were able to rinse their mouths independently. In terms of dietary consistency, regular diets were the most common (54.1%), followed by chopped diets (35.1%). Regarding denture status, 13 residents (35.1%) wore maxillary dentures, 14 (37.8%) wore mandibular dentures, and 10 (27.0%) routinely used dentures during meals.

Among the care workers, females accounted for the majority (90.0%), and most participants were care workers (90.9%). Regarding oral health education experience, 75.0% of care workers reported having received oral care education. Among those who had received education, the largest proportion had received education only once (40.0%).

### 3.2. Changes in Salivary IL-1β Levels Before and After the Care Worker-Centered Oral Health Intervention

**Figure 2:**
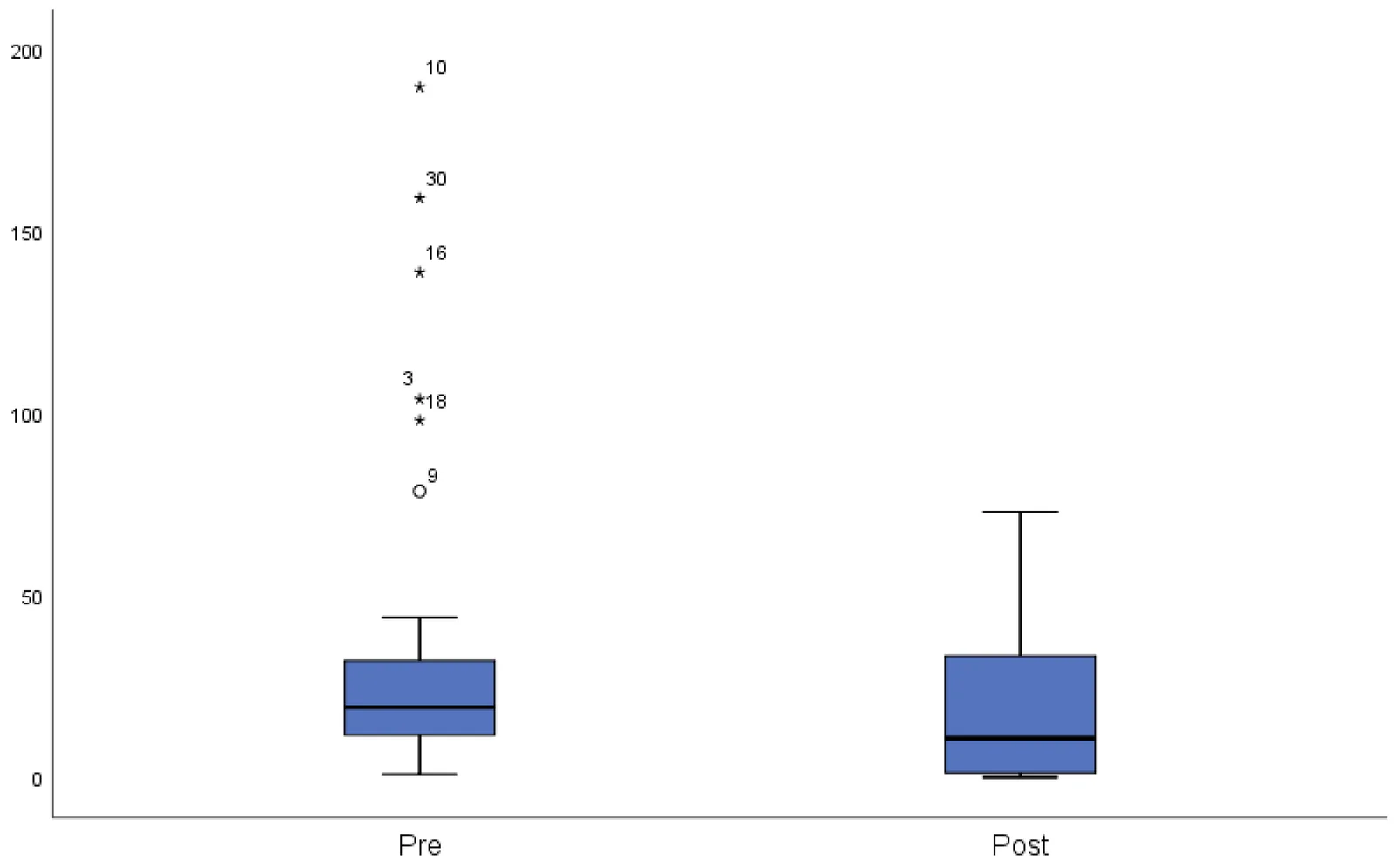
Distribution of salivary interleukin-1 beta (IL-1β) concentrations before and after the care worker-centered oral healthcare intervention. Circles indicate outliers, asterisks indicate extreme outliers, and the numbers adjacent to these symbols indicate the corresponding case numbers.

Changes in salivary IL-1β levels before and after the care worker-centered oral health intervention are presented in Table 2. Salivary IL-1β levels significantly decreased following the intervention. The median IL-1β level decreased from 19.33 pg/mL (interquartile range [IQR]: 11.49–34.66) at baseline to 10.78 pg/mL (IQR: 0.65–33.64) after the intervention (Z = −3.764, *p* = 0.001), with a large effect size (r = 0.62) (Figure 2).

### 3.3. Changes in Salivary IL-1β Levels According to Resident Characteristics

Table 3 presents the association between resident characteristics and changes in salivary IL-1β levels. Significant differences in changes in salivary IL-1β levels were observed according to long-term care grade (χ^2^ = 9.823, *p* = 0.044). The magnitude of reduction in IL-1β varied across care grades. The greatest reduction was observed in grade 1 residents (Δ = −36.37).

Changes in IL-1β levels also differed significantly according to communication ability (χ^2^ = 6.866, *p* = 0.032), with greater reductions observed among residents with preserved communication ability than among those with limited or absent communication ability.

Oral care refusal behavior was significantly associated with changes in IL-1β levels (χ^2^ = 9.488, *p* = 0.009). Residents without oral care refusal showed greater reductions in IL-1β, whereas those who refused oral care demonstrated minimal reductions or increased IL-1β levels.

## 4. Discussion

This study demonstrated that a care worker-centered oral healthcare program significantly reduced salivary IL-1β concentrations among older adults residing in LTCFs. These findings suggest that routine oral healthcare provided by trained care workers can effectively reduce the oral inflammatory burden in this population. Because IL-1β is a key pro-inflammatory cytokine involved in periodontal inflammation and tissue destruction, the observed reduction provides objective biological evidence of reduced oral inflammatory burden following the intervention. The observed reduction in salivary IL-1β was accompanied by a large effect size (r = 0.62), suggesting a substantial short-term reduction in oral inflammatory burden following the intervention.

Older adults living in LTCFs are widely recognized as having poor oral health and an increased risk of oral diseases owing to functional dependence, multimorbidity, and inadequate oral hygiene practices [6,21,25]. Previous intervention studies conducted in LTCFs have primarily focused on educating care workers in standardized toothbrushing techniques and denture care and have evaluated intervention outcomes using clinical indices, such as the plaque index, gingival index, and mucosal inflammation index [26,27]. Although these clinical indicators are well established, they are susceptible to examiner variability and may not fully reflect the biological inflammatory status of the oral cavity.

In contrast, the present study objectively evaluated the effectiveness of an oral healthcare intervention using salivary IL-1β, a biological marker of oral inflammation. Salivary IL-1β was selected because previous systematic reviews have consistently demonstrated that it is among the most extensively validated biomarkers of periodontal inflammation, with favorable diagnostic performance for periodontal disease and responsiveness to periodontal treatment [22,24,28,29]. As salivary IL-1β can be collected non-invasively, measured repeatedly, and is less influenced by examiner-dependent variability, it represents a promising biomarker for evaluating oral healthcare interventions in frail older adults, particularly in LTCFs, where comprehensive periodontal examinations are often difficult to perform.

The reduction in salivary IL-1β observed in this study may be attributed to improved daily oral hygiene practices delivered by trained care workers. Regular toothbrushing, denture care, oral moisturizing care, oral massage, and oral functional exercises likely reduced dental plaque accumulation and the oral microbial burden. Dental plaque is the primary etiologic factor initiating periodontal inflammation by stimulating the production of inflammatory cytokines [30], and improved plaque control has been associated with reduced expression of inflammatory mediators [31]. Therefore, the significant reduction in IL-1β observed after the intervention suggests that the care worker-centered oral healthcare program may have contributed to improved oral hygiene and reduced oral inflammatory activity. Although clinical periodontal parameters were not assessed, the observed biological reduction may nevertheless reflect improved oral hygiene conditions in this high-risk LTCF population, where access to professional oral healthcare is often limited.

Interestingly, the magnitude of IL-1β reduction differed according to residents’ functional and behavioral characteristics. Residents who were highly dependent on others for daily oral care showed greater reductions in IL-1β than those with higher levels of functional independence. Individuals with severe functional dependence generally experience poorer oral hygiene because they are unable to perform adequate self-care [32]. Consequently, they may derive greater benefit from structured oral healthcare provided by trained care workers, highlighting the importance of caregiver-assisted oral hygiene for this vulnerable population.

Conversely, residents with limited communication abilities or those who exhibited refusal behaviors during oral care showed little improvement or even increased salivary IL-1β concentrations. Communication impairment and care-resistant behaviors are common among older adults with dementia and cognitive impairment [33,34] and have consistently been identified as major barriers to effective oral healthcare delivery in LTCFs [35]. These findings indicate that the effectiveness of oral healthcare interventions is influenced by the quality of care provided as well as residents’ cognitive and behavioral characteristics. Accordingly, individualized oral healthcare strategies that accommodate residents’ functional status, communication abilities, and behavioral responses should be incorporated into future care programs. These findings suggest that functional dependence and care-resistant behaviors are important determinants of intervention effectiveness rather than merely baseline characteristics. It is also possible that some residents represented non-responders to the intervention, reflecting differences in baseline oral inflammatory status, underlying systemic conditions, adherence to oral care, or behavioral characteristics. Collectively, these findings highlight the need for individualized oral healthcare strategies that incorporate appropriate communication techniques and behavioral management approaches for residents with cognitive and functional impairments.

Although the differences were not statistically significant, residents who had difficulty maintaining mouth opening during oral care, were completely dependent on caregivers for daily oral hygiene, or had severe functional limitations showed smaller reductions in IL-1β. These findings further emphasize the need for care workers to acquire practical knowledge and skills that enable them to provide appropriate oral healthcare for residents with varying levels of physical and cognitive impairment. Previous studies have similarly reported that care workers frequently encounter care-resistant behaviors during oral care and perceive insufficient knowledge and practical skills as major barriers to providing appropriate oral healthcare [8,35]. Systematic oral healthcare education improves care workers’ knowledge, attitudes, and oral care performance [20,34], and many care workers express a willingness to participate in ongoing educational programs [36]. Together, these findings support the need for continuous professional education to enhance the quality of oral healthcare provided in LTCFs.

The present findings also have important implications for oral healthcare policy in long-term care settings. Although recent integrated community care policies in Korea have recognized oral healthcare as an essential service, many LTCFs continue to experience limited access to dental professionals. Under these circumstances, a care worker-centered oral healthcare program represents a practical and sustainable approach to improving residents’ oral health by strengthening the competencies of the existing workforce. Therefore, incorporating standardized oral healthcare education into routine care worker training may contribute to improving oral health and potentially supporting overall health among institutionalized older adults.

Several limitations should be acknowledged. First, the single-group quasi-experimental design without a control group limits causal inference. Second, the relatively small final sample size resulting from participant attrition may restrict the generalizability of the findings. In addition, the final analytical sample was smaller than the sample size estimated a priori, which may have reduced the statistical power to detect smaller intervention effects. Moreover, participant attrition may have introduced selection bias because many residents who were lost to follow-up experienced deterioration in their medical condition or died during the study period. Consequently, the final sample may not have fully represented the broader LTCF resident population, potentially reducing the variability of salivary IL-1β concentrations and influencing the estimated intervention effect. Third, the study was conducted in only two LTCFs, which may limit its applicability to other institutional settings. Fourth, oral inflammatory status was evaluated using a single salivary biomarker without concomitant clinical periodontal assessments. Although baseline oral health-related characteristics, including denture status, were collected, detailed clinical periodontal examinations (e.g., plaque accumulation, gingival inflammation, probing depth, and bleeding on probing) were not performed. Therefore, although the reduction in salivary IL-1β suggests decreased oral inflammatory activity, it was not possible to determine whether these biological changes were accompanied by clinically meaningful improvements in oral health. Furthermore, oral inflammatory status is influenced by multiple biological processes, and the use of a single biomarker may not fully capture the complexity of oral inflammation. Future studies should incorporate comprehensive clinical periodontal assessments together with multiple biological markers to provide a more complete evaluation of oral healthcare interventions. In addition, saliva samples were collected using two different methods according to residents’ ability to provide unstimulated saliva. Although this approach was necessary in this frail population, methodological heterogeneity may have influenced measured IL-1β concentrations. Future studies should employ standardized saliva collection protocols or analytical adjustments to minimize potential measurement bias. Finally, only the short-term effects of the 4-week intervention were assessed, and the long-term sustainability of the intervention remains unknown. Future multicenter randomized controlled trials incorporating both clinical periodontal assessments and salivary biomarkers are warranted to provide more comprehensive evidence regarding the effectiveness of care worker-centered oral healthcare interventions.

Despite these limitations, this study has several notable strengths. To our knowledge, this is one of the few studies to evaluate the effectiveness of a care worker-centered oral healthcare program using salivary IL-1β as an objective biological marker rather than relying solely on conventional clinical indices. These findings provide biological evidence supporting the effectiveness of oral healthcare interventions in LTCFs and offer a valuable framework for objectively evaluating future oral healthcare programs for institutionalized older adults.

## 5. Conclusions

In conclusion, a care worker-centered oral healthcare program significantly reduced salivary IL-1β concentrations among older adults residing in LTCFs, indicating reduced oral inflammatory burden. These findings support the potential role of care worker-delivered oral healthcare in reducing oral inflammatory burden among institutionalized older adults. Although salivary IL-1β appears to be a useful objective biomarker for evaluating oral healthcare interventions, larger controlled studies incorporating comprehensive clinical periodontal assessments and multiple biological markers with longer follow-up periods are required before these findings can be translated into routine practice or standard oral healthcare policy.

## Figures and Tables

**Table 1:** Baseline characteristics of the participants.

Participants	Variable	Group	*n* (%)
Residents (*n* = 37)	Sex	Male	14 (37.8)
		Female	23 (62.2)
	Long-term care grade	Grade 1	2 (5.4)
		Grade 2	4 (10.8)
		Grade 3	13 (35.1)
		Grade 4	15 (40.5)
		Grade 5	3 (8.1)
	Oral hygiene frequency	≤1 time/day	3 (8.1)
		≥2 times/day	34 (91.9)
	Use of oral hygiene aids	Toothbrush	32 (86.5)
		Interdental brush	0 (0.0)
		Denture cleaner	12 (32.4)
		Dental floss	0 (0.0)
		None	23 (62.2)
	Mouth rinsing ability	Able	26 (70.3)
		Unable	11 (29.7)
	Diet consistency	Regular diet	20 (54.1)
		Minced diet	2 (5.4)
		Soft minced diet	13 (35.1)
		Liquid diet	2 (5.4)
	Denture	Maxillary denture	13 (35.1)
		Mandibular denture	14 (37.8)
		Denture use during meals	10 (27.0)
Care workers (*n* = 40)	Sex	Male	4 (10.0)
		Female	36 (90.0)
	Occupation	Care worker	36 (90.0)
		Nurse aide	1 (2.5)
		Nurse	3 (7.5)
	Educational Experience	Yes	30 (75.0)
		No	10 (25.0)
	Number of previous oral healthcare education sessions	One	12 (40.0)
		Two	10 (33.3)
		Three or more	8 (26.7)

Data are presented as *n* (%).

**Table 2:** Changes in salivary IL-1β concentrations before and after the care worker-centered oral healthcare intervention.

Cytokine	Pre-Intervention	Post-Intervention	Z	*p*-Value	Effect Size (r)
IL-1β (pg/mL)	19.33 (11.49–34.66)	10.78 (0.65–33.64)	−3.764	0.001	0.62

Data are presented as median (interquartile range). Because the data were not normally distributed, changes in salivary interleukin-1 beta (IL-1β) before and after the intervention were analyzed using the Wilcoxon signed-rank test. A two-sided *p*-value < 0.05 was considered statistically significant.

**Table 3:** Association between resident characteristics and changes in salivary IL-1β levels.

Variable	Group	*n*	IL-1β		ΔIL-1β (Median, IQR)	*p*	Statistic
			Pre	Post			
Sex	Male	14	18.02 ± 11.65	12.24 ± 15.07	−6.53 (−14.16–2.25)	0.175	Z = −1.378
	Female	23	46.89 ± 54.09	22.31 ± 24.61	−12.36 (−45.27–−2.80)		
Age (years)	65–69	2	29.27 ± 11.36	27.47 ± 9.09	−1.80	0.807	χ^2^ = 1.610
	70–79	4	42.02 ± 43.37	18.80 ± 17.61	−21.18 (−55.05–6.56)		
	80–89	22	36.58 ± 43.30	18.65 ± 22.78	−12.95 (−27.08–−0.22)		
	≥90	9	33.25 ± 59.09	16.02 ± 25.08	−10.03 (−18.43–−0.34)		
Long-term care grade	1	2	71.02 ± 38.28	34.65 ± 5.91	−36.37	0.044	χ^2^ = 9.823
	2	4	16.07 ± 16.60	15.06 ± 15.26	−0.22 (−2.61–−0.18)		
	3	13	39.64 ± 52.44	26.67 ± 26.43	−4.61 (−29.90–10.75)		
	4	15	28.91 ± 45.10	10.62 ± 18.29	−12.36 (−20.46–−4.04)		
	5	3	58.43 ± 39.67	16.32 ± 22.07	−32.69		
Communication ability	Unable	8	46.38 ± 47.08	31.94 ± 23.54	−10.96 (−48.07–12.60)	0.032	χ^2^ = 6.866
	Limited	13	13.03 ± 6.66	9.42 ± 11.24	−4.04 (−10.83–0.69)		
	Able	16	49.39 ± 55.83	19.16 ± 24.82	−22.48 (−42.13–−5.65)		
Mobility	Unable	18	26.98 ± 35.55	15.59 ± 20.84	−6.52 (−24.80–1.09)	0.257	χ^2^ = 2.718
	Limited	11	38.13 ± 44.41	21.26 ± 22.16	−12.36 (−20.46–−0.20)		
	Independent	8	53.20 ± 63.59	21.25 ± 25.55	−13.76 (−55.05–−5.11)		
Ability to perform daily oral care	Independent	5	73.06 ± 73.89	31.87 ± 28.73	−25.73 (−89.85–−0.25)	0.369	χ^2^ = 1.997
	Requires assistance	21	29.90 ± 38.59	13.04 ± 18.45	−12.36 (−27.81–−0.20)		
	Fully dependent	11	30.68 ± 37.17	22.85 ± 23.06	−9.67 (−13.48–−0.20)		
Refusal of oral care	None	20	45.37 ± 50.15	20.32 ± 22.76	−13.40 (−32.30–−9.76)	0.009	χ^2^ = 9.488
	Occasionally	9	35.12 ± 48.87	15.32 ± 25.31	−8.43 (−41.88–−1.50)		
	Always	8	13.40 ± 9.66	17.53 ± 17.02	0.97 (−3.09–13.49)		
Ability to keep the mouth open during oral care	Unable	7	21.93 ± 6.21	22.73 ± 18.85	−0.20 (−14.52–16.87)	0.111	χ^2^ = 4.405
	Difficult	9	33.13 ± 49.78	16.75 ± 24.33	−4.61 (−32.46–0.69)		
	Able	21	41.85 ± 50.58	17.84 ± 22.51	−13.31 (−31.91–−3.11)		
Ability to rinse	Unable	11	30.01 ± 36.90	22.85 ± 23.64	−4.04 (−14.52–9.53)	0.973	χ^2^ = 0.055
	Able	26	38.48 ± 48.67	16.66 ± 21.24	−12.19 (−31.52–−2.33)		
Hypertension	No	11	48.55 ± 58.90	21.04 ± 27.93	−13.31 (−32.69–−4.04)	0.192	Z = −1.329
	Yes	26	30.64 ± 38.06	17.43 ± 19.25	−9.23 (−16.01–−0.04)		
Diabetes mellitus	No	22	29.43 ± 42.08	17.51 ± 22.94	−9.05 (−12.96–−0.04)	0.105	Z = −1.640
	Yes	15	45.55 ± 49.19	19.95 ± 20.79	−20.46 (−32.69–−0.89)		
Dementia	No	7	16.17 ± 13.40	10.93 ± 13.61	−3.41 (−12.02–3.37)	0.172	Z = −1.396
	Yes	30	40.58 ± 48.75	20.27 ± 23.16	−12.48 (−27.47–−0.23)		
Osteoporosis	No	34	34.33 ± 17.29	18.13 ± 22.41	−9.85 (−24.80–−0.19)	0.165	Z = −1.447
	Yes	3	54.49 ± 42.87	22.64 ± 16.36	−20.46		

Pre- and post-intervention IL-1β concentrations are presented as mean ± standard deviation for descriptive purposes. ΔIL-1β was calculated as the post-intervention value minus the pre-intervention value and is presented as the median (interquartile range). Between-group differences in ΔIL-1β were analyzed using the Mann–Whitney U test for two-group comparisons or the Kruskal–Wallis test for comparisons involving three or more groups. A two-sided *p*-value < 0.05 was considered statistically significant.

## Data Availability

The data presented in this study are available from the corresponding author upon reasonable request. The data are not publicly available due to privacy and ethical restrictions involving older adults residing in long-term care facilities.
